# Emerging Roles of Activity-Dependent Alternative Splicing in Homeostatic Plasticity

**DOI:** 10.3389/fncel.2020.00104

**Published:** 2020-05-12

**Authors:** Agnes Thalhammer, Fanny Jaudon, Lorenzo A. Cingolani

**Affiliations:** ^1^Center for Synaptic Neuroscience and Technology, Istituto Italiano di Tecnologia (IIT), Genoa, Italy; ^2^IRCCS Ospedale Policlinico San Martino, Genoa, Italy; ^3^Department of Life Sciences, University of Trieste, Trieste, Italy

**Keywords:** alternative splicing, homeostatic plasticity, repressor element 1 silencing transcription factor (REST), homer1, P/Q-type Ca^2+^ channels

## Abstract

Homeostatic plasticity refers to the ability of neuronal networks to stabilize their activity in the face of external perturbations. Most forms of homeostatic plasticity ultimately depend on changes in the expression or activity of ion channels and synaptic proteins, which may occur at the gene, transcript, or protein level. The most extensively investigated homeostatic mechanisms entail adaptations in protein function or localization following activity-dependent posttranslational modifications. Numerous studies have also highlighted how homeostatic plasticity can be achieved by adjusting local protein translation at synapses or transcription of specific genes in the nucleus. In comparison, little attention has been devoted to whether and how alternative splicing (AS) of pre-mRNAs underlies some forms of homeostatic plasticity. AS not only expands proteome diversity but also contributes to the spatiotemporal dynamics of mRNA transcripts. Prominent in the brain where it can be regulated by neuronal activity, it is a flexible process, tightly controlled by a multitude of factors. Given its extensive use and versatility in optimizing the function of ion channels and synaptic proteins, we argue that AS is ideally suited to achieve homeostatic control of neuronal output. We support this thesis by reviewing emerging evidence linking AS to various forms of homeostatic plasticity: homeostatic intrinsic plasticity, synaptic scaling, and presynaptic homeostatic plasticity. Further, we highlight the relevance of this connection for brain pathologies.

## Introduction: From Genes to Function

**Figure 1:**
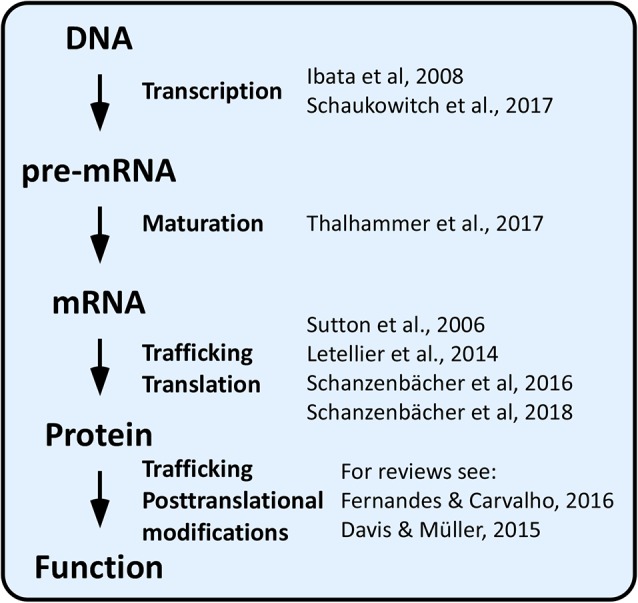
Genes to function in homeostatic plasticity.

Over the last two decades, a vast array of homeostatic plasticity adaptations, which enable neuronal networks to stabilize their activity in the face of external perturbations, have been identified. These involve adjustments in synaptic strength by means of pre- and postsynaptic mechanisms (homeostatic synaptic plasticity) and in intrinsic excitability (homeostatic intrinsic plasticity). Ultimately, both synaptic and intrinsic forms of homeostatic plasticity depend on changes in expression or activity of ion channels and synaptic proteins, which may occur at the gene, transcript, or protein level (Figure 1).

By far, the most extensively investigated homeostatic mechanisms involve changes in protein function or localization by means of posttranslational modifications affecting protein–protein interactions and trafficking (reviewed in Turrigiano, 2011; Davis and Müller, 2015; Fernandes and Carvalho, 2016; Cingolani et al., 2019).

Chronic changes in network activity can also be counteracted by regulating protein translation. For example, increased surface expression of the GluA1 subunit of AMPA-type glutamate receptors (AMPARs) compensates blockade of network activity within a few hours. This form of homeostatic synaptic plasticity, known as synaptic upscaling, requires local protein synthesis because it is prevented by dendritic application of the protein synthesis inhibitors anisomycin or emetine (Sutton et al., 2006), and it involves downregulation of miR92a (Letellier et al., 2014; Dubes et al., 2019). Further, the transcription of hundreds of genes was recently shown to be up- or downregulated at early (2 h) and late (24 h) stages of the homeostatic response (Schanzenbächer et al., 2016, 2018).

Synaptic upscaling following tetrodotoxin (TTX)-induced suppression of network activity is dependent also on gene transcription because the transcription inhibitor actinomycin D (ActD) blocks effectively upscaling of miniature excitatory postsynaptic currents (mEPSCs) and dendritic accumulation of the AMPAR subunit GluA2 (Ibata et al., 2008). More recently, chronic suppression of network activity was shown to alter the transcription of tens of genes, including that for the AMPAR clustering protein neuronal pentraxin-1 (*Nptx1*); Ca^2+^ entry *via* T-type voltage-gated Ca^2+^ channels (VGCCs) appears essential for this signaling pathway (Schaukowitch et al., 2017). Conversely, chronic augmentation of network activity leads to Ca^2+^-dependent changes in the expression of hundreds of genes (Flavell and Greenberg, 2008; Schaukowitch et al., 2017), some of which, such as brain-derived neurotrophic factor (BDNF), calcineurin, and MeCP2, are known players in homeostatic synaptic plasticity (Fernandes and Carvalho, 2016). Neuronal activity also increases the expression levels of immediate early genes, such as Arc (aka Arg3.1), which induces a counterbalancing internalization of AMPARs (Shepherd et al., 2006) and, when localized in the nucleus, decreases transcription of the AMPAR subunit GluA1, thereby reducing synaptic strength (Korb et al., 2013).

In comparison to the above outlined molecular mechanisms, little attention has thus far been devoted to whether and how homeostatic adaptations are achieved at the level of alternative splicing (AS) of pre-mRNAs (Figure 1). As detailed below, this lack of attention may come as a surprise because some AS events are well-known for being controlled by neuronal activity and because AS is ideally suited to optimize protein function to new challenges (Raj and Blencowe, 2015; Vuong et al., 2016; Baralle and Giudice, 2017). Here, we review recent findings linking homeostatic plasticity to AS and discuss the relevance of activity-dependent AS to achieve homeostatic control of neuronal output in health and diseased states.

## Alternative Splicing

During RNA maturation, intervening noncoding RNA sequences (introns) are removed while coding sequences (exons) are joined together, thus contributing to transforming a newly transcribed mRNA (pre-mRNA) into a mature mRNA. RNA splicing is performed by a multi-molecular RNA–protein complex, the spliceosome, which binds to specific sequences on the pre-mRNA. These include a donor site (5^′^ end of the excised intron), an acceptor site (3^′^ end of the intron), and, upstream of the 3^′^ site, a polypyrimidine tract and a branch point. For some genes, rather than being univocal, the splicing process creates a range of mature mRNAs, each with a unique exon composition. If translated, these mRNA splice isoforms will produce multiple protein variants with potentially distinct functions. We talk in this case of AS. AS is regulated by *cis*-acting elements (regulatory RNA sequences), which act as splicing enhancers or repressors by recruiting *trans*-acting splicing factors (proteins or ribonucleoproteins) that favor or inhibit different steps of the splicing reaction (Matera and Wang, 2014).

In higher eukaryotes, AS has the potential to convert a limited number of genes into an astounding variety of proteins depending on developmental stage, brain region, and cell types. For example, thousands of mRNA splicing isoforms were found to be different between neurons and glial cells when comparing purified brain cell populations (Zhang et al., 2014). Indeed, some splicing factors display cell-type specific expression (Nguyen et al., 2016; Furlanis and Scheiffele, 2018), while others regulate specific splicing events during brain development (Norris and Calarco, 2012). Transcriptomic and proteomic studies indicate that more than 90% of mammalian genes undergo AS, with the brain exhibiting the most complex repertoire of splice variants (Pan et al., 2008; Wang et al., 2008; Kim et al., 2014; Schreiner et al., 2015). In some cases, as for neurexins and calcium channels, one single gene can give rise to potentially thousands of different mRNA isoforms (Ullrich et al., 1995; Soong et al., 2002; Lipscombe et al., 2013; Schreiner et al., 2014; Treutlein et al., 2014), many of which have been identified at the protein level (Kim et al., 2014; Schreiner et al., 2015). It should also be noted that AS is not limited to diversifying the coding sequence of an mRNA but can also modify the selection of 5’ and 3’ untranslated regions (UTRs), thus affecting stability, subcellular localization and translation of mRNAs (Hermey et al., 2017; Mauger and Scheiffele, 2017).

In order to be instructive for homeostatic plasticity, AS needs to fulfill two criteria: (i) it must be regulated by neuronal activity; and (ii) the outcome of the splicing process must result in a homeostatic compensation. We will explore the requirement of AS in homeostatic plasticity in the next paragraphs following three exemplary cases.

## NSR100, Microexons, and Rest in Homeostatic Plasticity

**Figure 2:**
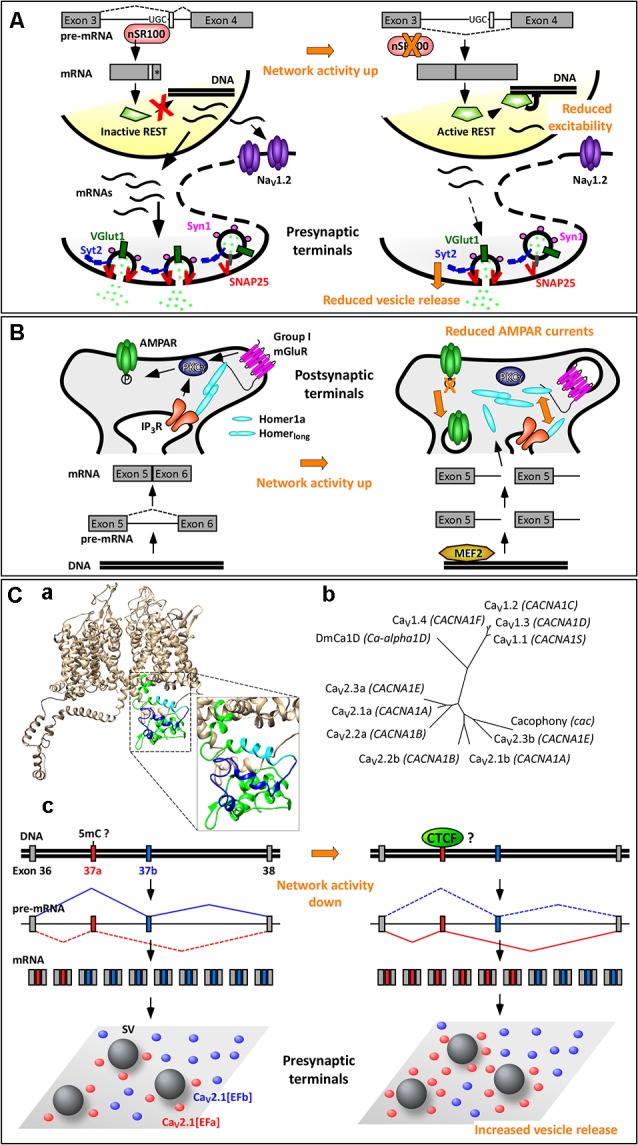
Activity-dependent alternative splicing in homeostatic plasticity. **(A)** A chronic increase in neuronal activity downregulates the expression of the splicing factor nSR100, with consequent skipping of a 16-nt-long microexon in the pre-mRNA of the transcriptional repressor REST (repressor element 1 silencing transcription factor). The resulting REST protein is active and reduces the expression of Na_V_1.2 and of presynaptic proteins. These two effects contribute to homeostatic intrinsic plasticity and presynaptic homeostatic plasticity, respectively. (*) Indicates a STOP codon. **(B)** The selective induction of the short isoform Homer1a upon increase in neuronal activity is mediated by the transcription factor myocyte enhancer factor 2 (MEF2), which promotes expression of the Homer1 gene, and by a concomitant termination of transcription between exons 5 and 6. Homer1a outcompetes the longer isoforms of Homer1, resulting in dispersion of group 1 mGluRs and dephosphorylation of AMPARs. This contributes to synaptic downscaling. **(C)** Mutually exclusive splicing of P/Q-type Ca^2+^ channels in presynaptic homeostatic plasticity. **(Ca)** Structural model of human Ca_V_2.1[EFb] (UniProt ID: O00555; Martinez-Ortiz and Cardozo, 2018), highlighting the full C-terminus (green, cyan, blue), the part of the EF-hand-like domain shared between Ca_V_2.1[EFa] and Ca_V_2.1[EFb] (E helix; cyan) and the sequence specific to Ca_V_2.1[EFb] (loop, F helix and downstream residues; blue). **(Cb)** Phylogenetic tree of human Ca_V_1 and Ca_V_2 channels and of Cacophony and DmCa1D from *Drosophila melanogaster* for the amino acidic region corresponding to exons 37 of Ca_V_2.1 (Clustal Omega www.ebi.ac.uk/Tools/msa/clustalo/, rendering using TreeDyb, http://www.phylogeny.fr/one_task.cgi?tasktype=treedyn, Chevenet et al., 2006); UniProt IDs: Ca_V_1.1: Q13698, aa: 1414–1446; Ca_V_1.2: Q13936, aa: 1587–1589; Ca_V_1.3: Q01668, aa: 1497–1529; Ca_V_1.4: O60840, aa: 1474–1506; Ca_V_2.1b: O00555, aa: 1843–1875; Ca_V_2.2b: Q00975, aa: 1741–1773; Ca_V_2.3b: Q15878, aa: 1756–1788; Ca_V_2.1a: O00555-4, aa: 1844–1876; Cacophony: P1645, aa: 1370–1402; DmCa1D: Q24270, aa: 1959–1991; sequences for Ca_V_2.2a and Ca_V_2.3a are as in Thalhammer et al. (2017). The three exons 37a cluster together as do the three exons 37b, suggesting conservation of these mutually exclusive exons across Ca_V_2 channels; the corresponding region of Cacophony from *D. melanogaster* is more tightly related to exon 37b. **(Cc)** The increased expression of the isoform Ca_V_2.1[EFa] upon chronic activity deprivation might occur following demethylation of the exon 37a locus with consequent binding of the chromatin organizer CCCTC-binding factor (CTCF) to it. Ca_V_2.1[EFa] localizes in close proximity to fuse-competent synaptic vesicles, thereby supporting effectively vesicle release and presynaptic homeostatic plasticity. Drawing of relative exon/intron length is to scale only in (**Cc**); numbers of mRNAs and proteins are not intended to be quantitative.

Some splicing factors, such as Nova-1/2, Rbfox-1/2/3, Ptbp1/2, and nSR100 are highly enriched in neurons. Among these, the Ser/Arg repeat-related protein of 100 kDa (nSR100, aka SRRM4) binds to intronic enhancer UGC elements close to the 3’ splice sites to promote microexon inclusion (Figure 2A; Raj and Blencowe, 2015). Microexons are a class of cassette exons (exons that can be included or not in the mature transcript) that tend to be located in surface loops and intrinsically disordered regions. They generally have a length of 9–21 nucleotides (nt), often in multiples of three nt, hence leading to alternative versions of a protein with altered functions, protein–protein interaction motifs, or posttranslational modifications. Microexons are especially important in the brain, where they constitute nearly one third of all neural-regulated splicing events. They are frequently misregulated in the brain of individuals with autism spectrum disorder; this is likely due to increased neuronal activity, often associated with autism spectrum disorder, resulting in a rapid decrease in nSR100 expression and increased skipping of microexons (Irimia et al., 2014; Quesnel-Vallières et al., 2016).

Although generally frame preserving, microexon inclusion promoted by nSR100 can also disrupt the reading frame of a gene. For example, one well-known downstream target of nSR100 is the transcriptional repressor REST (repressor element 1 silencing transcription factor; aka NRSF, neural restrictive silencing factor), which silences a multitude of neural genes (Raj et al., 2011). In this case, nSR100 promotes the inclusion of a 16-nt-long microexon located between the third and fourth exons, leading to a frameshift introducing a stop codon at the beginning of the fourth exon. The resulting isoform, REST4, is truncated and lacks the domains required for transcriptional repression of target genes (Raj et al., 2011). When neuronal activity increases, nSR100 expression is rapidly downregulated (Quesnel-Vallières et al., 2016), resulting in skipping of the 16-nt-long microexon and production of the active isoform of REST (Figure 2A). Accordingly, REST is upregulated in primary neuronal cultures after 48–96 h of network hyperactivity, and this decreases the expression of its targets, including the sodium channel Na_V_1.2, the calcium channel Ca_V_3.2, and various presynaptic proteins (SNAP-25, Synapsin-1, Synaptotagmin-2, and vGlut-1; van Loo et al., 2012; Pozzi et al., 2013). Downregulation of Na_V_1.2 makes it more difficult for a neuron to elicit action potentials, thus contributing to homeostatic intrinsic plasticity (Pozzi et al., 2013). Decreased expression of presynaptic proteins correlates with a reduction in the number of docked synaptic vesicles and in the frequency of mEPSCs, thus contributing to presynaptic homeostatic plasticity, a prominent form of homeostatic synaptic plasticity (Figure 2A; Pecoraro-Bisogni et al., 2018).

## Alternative Splice Isoforms of HOMER1 in Synaptic Scaling

The Homer1 gene generates long and short splice isoforms. The major isoforms, Homer1b, Homer1c, and Homer1d, are long, constitutively expressed, and act as scaffold proteins at postsynaptic sites (Fagni et al., 2002; Shiraishi-Yamaguchi and Furuichi, 2007). In response to various stimuli, such as electroconvulsive seizures, cocaine, kainate or nicotine exposure, two truncated isoforms of Homer1, Homer1a and Ania3, which have all the characteristics of immediate early gene products, are rapidly (1–4 h) induced (Brakeman et al., 1997; Kato et al., 1997; Berke et al., 1998; Bottai et al., 2002). This is due to myocyte enhancer factor 2 (MEF2) family transcription factors, which boost transcription of the Homer1 gene and to a concomitant termination of transcription within the large central intron between exons 5 and 6, leading to use of alternative poly(A) sites. Because of this coordinated increase in transcription rate and premature transcription termination, only the short isoforms of Homer1 are induced by neuronal activity (Figure 2B; Bottai et al., 2002; Flavell et al., 2008).

The long isoforms of Homer1 consist of two major domains: (i) an N-terminal Enabled/Vasp homology 1 (EVH1) domain, which binds to proline-rich sequences in Group 1 metabotropic glutamate receptors (mGluR1 and 5), inositol-1,4,5-trisphosphate (IP_3_) receptors, ryanodine receptors, TRPC1 ion channels, and the scaffold protein Shank; and (ii) a C-terminal coiled-coil (CC) structure followed by leucine zipper motifs, which favor oligomerization of homer proteins (Szumlinski et al., 2006). The long isoforms of Homer1 are therefore essential in cross-linking multiple postsynaptic proteins. Conversely, the short isoforms of Homer1 lack the C-terminal domain involved in oligomerization; once induced, they act as dominant-negative regulators disrupting the binding between Homer1 long isoforms and their effectors (Xiao et al., 1998; Kammermeier and Worley, 2007).

Increasing network activity, therefore, upregulates transiently the expression of Homer1a, which, among other things, disrupts the protein–protein interactions clustering group 1 mGluRs at perisynaptic sites. In addition, Homer1a acts as an endogenous allosteric modulator of mGluR1/5; that is, it supports a glutamate-independent activity of these mGluRs (Ango et al., 2001). This is essential in promoting homeostatic downscaling of synaptic AMPARs both *in vitro* and *in vivo* (Figure 2B; Hu et al., 2010; Diering et al., 2017), as reviewed elsewhere in this topic (Cingolani et al., 2019).

The expression of Homer1a is increased in the CA1 region of the hippocampus in schizophrenic patients (Matosin et al., 2016) and up- or downregulated in different brain regions of patients with either bipolar disorder or major depression (Leber et al., 2017). Furthermore, in *Fmr1* knockout mice, a model for fragile X syndrome, mGluR5 is preferentially associated to Homer1a, leading to an enhanced glutamate-independent activation of this receptor and consequent neocortical circuit dysfunctions and behavioral abnormalities. Some of these defects are rescued by genetic deletion of Homer1a (Giuffrida et al., 2005; Ronesi et al., 2012), which is consistent with brain function being dependent on an appropriate ratio between short and long isoforms of Homer1.

## Alternative Splicing of P/Q-Type Ca^2+^ Channels in Presynaptic Homeostatic Plasticity

Mutually exclusive splicing is a form of AS, whereby the splicing of two or more exons is coordinated in such a way that only one is retained while the others are spliced out from the mature mRNA (Figure 2Cc). Mutually exclusive exons are generally highly similar possibly because they originated from exon duplication. However, far from being redundant, they usually allow the formation of protein isoforms that differ in the function of specific domains while preserving the overall structure and size. Indeed, mutually exclusive splicing in many genes is spatially and temporally regulated (Pohl et al., 2013). Recent data indicate that mutually exclusive exons may be much more frequent in mammals than previously thought and that they are overrepresented in genes encoding for ion channels (Hatje et al., 2017). Interestingly, the occurrence of pathogenic single-nucleotide polymorphisms (SNPs) in mutually exclusive and cassette exons is significantly higher than in other types of exons, suggesting that these two forms of AS are especially susceptible to pathogenic mutations. For mutually exclusive splicing, the pathogenic SNPs tend to be present in only one of the two possible exons. Thus, the second mutually exclusive exon cannot normally replace the defective one either because of functional diversification or because of differential spatiotemporal expression patterns (Hatje et al., 2017).

A well-characterized case of mutually exclusive exons occurs in the proximal C-terminus of the pore-forming α_1_ subunit of the Ca_V_2 VGCCs (Ca_V_2.1, Ca_V_2.2, and Ca_V_2.3; Bourinet et al., 1999; Bell et al., 2004; Gray et al., 2007; Hatje et al., 2017), which serve as primary Ca^2+^ entry for the release of synaptic vesicles at most presynaptic terminals. The 97-nt-long mutually exclusive exons 37a and 37b encode part of an EF-hand-like domain, thus creating two variants of it (EFa and EFb; Figure 2Ca; Bourinet et al., 1999; Chaudhuri et al., 2004; Thalhammer et al., 2017). This motif is not specific to Ca_V_2 channels but conserved across Ca^2+^ and Na^+^ channels (Babitch, 1990; Ben-Johny et al., 2014); in particular, exons 37a and 37b in Ca_V_2 channels exhibit a high level of similarity with the corresponding exons in Ca_V_1 channels (Figure 2Cb).

Which are the functions of the EF-hand-like domain and why do Ca_V_2 channels need two variants of it? Three major, not mutually exclusive, functional differences have been proposed. In N-type Ca^2+^ channels (Ca_V_2.2), mutually exclusive splicing at exons 37 has been shown to regulate sensitivity of the channel to voltage-independent inhibition by G protein-coupled receptors (GPCRs). That is, several GPCRs, including opioid receptors, inhibit Ca_V_2.2[EFa] but not Ca_V_2.2[EFb] through kinase phosphorylation of a tyrosine residue (Y1743) present exclusively in the former isoform (Raingo et al., 2007; Andrade et al., 2010). Because Ca_V_2.2[EFa] is enriched in capsaicin-responsive nociceptors of dorsal root ganglia (Bell et al., 2004), this isoform-specific regulation mediates analgesia, for example, by morphine (Andrade et al., 2010).

In P/Q-type Ca^2+^ channels (Ca_V_2.1), the two isoforms have been shown to differ in how elevations in intracellular Ca^2+^ regulate the activity of the channel. Specifically, activation of Ca_V_2.1[EFa], but not Ca_V_2.1[EFb], is facilitated by preceding Ca^2+^ entry (Ca^2+^-dependent facilitation, CDF; Chaudhuri et al., 2004). This is in accordance with a large body of evidence indicating that the EF-hand-like domain in the proximal C-terminus of Ca^2+^ and Na^+^ channels, rather than binding directly to Ca^2+^, represents a general transduction element for the regulation of the channel by Ca^2+^-calmodulin (Peterson et al., 2000; Ben-Johny et al., 2014; Gardill et al., 2018). Calmodulin itself binds, in a Ca^2+^-independent manner, to downstream domains in the C-terminus of Ca^2+^ and Na^+^ channels (Peterson et al., 1999; Zuhlke et al., 1999; Mori et al., 2000; Erickson et al., 2001).

More recently, experiments in native systems have revealed that the two isoforms of Ca_V_2.1 regulate neurotransmitter release and short-term synaptic plasticity at hippocampal synapses in opposite directions. While Ca_V_2.1[EFa] promotes synaptic efficacy and short-term synaptic depression, Ca_V_2.1[EFb] characterizes synapses with low release probability and prominent short-term synaptic facilitation (Thalhammer et al., 2017). This is contrary to what the isoform-specific CDF, as characterized in non-neuronal cells, would have predicted (Chaudhuri et al., 2004; Weyrer et al., 2019); it likely reflects instead a differential spatial relationship of the two isoforms to fuse-competent synaptic vesicles, with a tight and loose coupling configuration for Ca_V_2.1[EFa] and Ca_V_2.1[EFb], respectively (Figure 2Cc; Thalhammer et al., 2017). More in general, AS of Ca_V_2.1 might underlie most of the intra- and inter-synaptic differences in nanoscale topographical arrangements of this channel, as recently revealed (Holderith et al., 2012; Nakamura et al., 2015; Rebola et al., 2019).

Whereas the expression of Ca_V_2.1[EFb] remains relatively constant throughout postnatal development, that of Ca_V_2.1[EFa] increases postnatally, in parallel with a tightening of the coupling between VGCCs and the neurotransmitter release machinery. As a result, both isoforms are expressed at similar levels in most regions of the adult brain (Bourinet et al., 1999; Soong et al., 2002; Vigues et al., 2002; Chaudhuri et al., 2004; Thalhammer et al., 2017). The developmental upregulation of Ca_V_2.1[EFa] occurs in rodents between the second and third postnatal week, the same period when ataxic symptoms become apparent in Ca_V_2.1^−/−^ knockout mice (Mark et al., 2011). Further, four point mutations associated with episodic ataxia type II have been identified in the exon 37a of *CACNA1A* (the gene for the α_1_ subunit of Ca_V_2.1) in four unrelated families (Graves et al., 2008; Mantuano et al., 2010), while none has been found, to date, in exon 37b, suggesting that Ca_V_2.1[EFa] might be more relevant for the etiology of episodic ataxia type II than Ca_V_2.1[EFb].

At the cellular level, while most neurons express both isoforms to various degrees, parvalbumin interneurons, which rely on P/Q-type Ca^2+^ channels to form synapses characterized by nanodomain coupling, high release probability, and short-term synaptic depression (Eggermann et al., 2012), stand out for expressing exclusively Ca_V_2.1[EFa] (Huntley et al., 2020), again pointing to functional synaptic specialization of the two Ca_V_2.1 splice isoforms.

Besides these differences in spatiotemporal expression patterns, the relative synaptic abundance of the two isoforms is regulated by network activity in a homeostatic fashion. Specifically, hippocampal neurons increase exclusively the synaptic expression of Ca_V_2.1[EFa] in response to activity deprivation. Because this isoform is the more efficient of the two in driving vesicle release, its higher expression levels appear perfectly suited to counteract the decrease in network activity (Figure 2Cc; Thalhammer et al., 2017). These findings provide therefore a precise molecular basis for the involvement of P/Q-type Ca^2+^ channels in presynaptic homeostatic plasticity (Frank et al., 2006; Jakawich et al., 2010; Lazarevic et al., 2011; Zhao et al., 2011; Jeans et al., 2017) and highlight the importance of activity-dependent AS in homeostatic synaptic plasticity.

Although it is not known how network activity regulates this splicing event, it has recently been proposed that inclusion of exon 37a or 37b in Ca_V_2.2 is consequent to differences in chromatin structure and transcription rates, rather than being directly regulated at the mRNA level (Javier et al., 2019; Lopez Soto and Lipscombe, 2020). Because splicing occurs mostly co-transcriptionally (Luco et al., 2011), rapid transcription of *Cacna1b* (the gene for the α_1_ subunit of Ca_V_2.2) would lead to simultaneous availability to the splicing machinery of the two mutually exclusive exons. Direct competition between them would results in inclusion of the downstream stronger exon 37b. Conversely, a slow transcription rate would favor recruitment of the splicing machinery to the first upstream exon 37a, thus leading to inclusion of this weaker exon into the final transcript. Indeed, the zinc finger DNA-binding protein CCCTC-binding factor (CTCF), a well-known organizer of chromatin architecture, binds to the exon 37a locus of *Cacna1b* to promote inclusion of this exon (Javier et al., 2019; Lopez Soto and Lipscombe, 2020). This is likely because CTCF favors the formation of intragenic chromatin loops and slows down the elongation rate of the RNA polymerase II (Pol II; Shukla et al., 2011; Ruiz-Velasco et al., 2017). Importantly, CTCF binding is not constitutive but prevented by methylation of the *Cacna1b* exon 37a locus, which consequently leads to exon 37a exclusion (Javier et al., 2019; Lopez Soto and Lipscombe, 2020).

Because the methylation level of chromosomal DNA is key to both memory formation and homeostatic synaptic plasticity (Day and Sweatt, 2010; Meadows et al., 2015), it is conceivable that activity-dependent methylation and demethylation might regulate also the inclusion of exon 37a in *Cacna1a* during presynaptic homeostatic plasticity. According to databases of chromatin immunoprecipitation followed by sequencing (ChIP-seq^1^, ENCODE Project Consortium, 2012), CTCF binds indeed also to the *Cacna1a* exon 37a locus (Figure 2Cc).

## Discussion: Implications of Activity-Dependent Alternative Splicing for Brain Disorders

Genome-wide transcriptomic studies indicate that AS is more prominent in the brain than in other tissues (Yeo et al., 2004; Pan et al., 2008). Accordingly, defects in AS have been implicated in neurological and neurodegenerative disorders (Raj and Blencowe, 2015; Furlanis and Scheiffele, 2018; Montes et al., 2019). AS defects can originate from mutations that alter either *cis*-acting elements on specific genes or *trans*-acting splicing factors affecting the splicing of multiple transcripts. As discussed briefly in this minireview article, the former mutations are prominent in mutually exclusive and cassette exons involved mostly in monogenic brain pathologies such as episodic ataxia type II, the latter are especially critical for multifactorial brain disorders, for example, for autism spectrum disorder (Gehman et al., 2011; Voineagu et al., 2011; Irimia et al., 2014; Quesnel-Vallières et al., 2016; Gonatopoulos-Pournatzis et al., 2018).

In both cases, to fully understand how defective AS alters circuit and brain function, it is important to consider that some AS events in the brain are regulated by network activity and that the outcome of the splicing process can in turn compensate for changes in activity levels, thus establishing negative feedback loops that make brain function especially resilient to damage. Rather than being direct, the effects of defective AS on brain function are therefore likely to be indirectly mediated by deficient or aberrant homeostatic plasticity mechanisms.

Elucidating the interplay between activity-dependent AS and homeostatic plasticity, as well as implementing new technologies, such as genome editing approaches aimed at correcting pathogenic mutations interfering with AS or at rebalancing splice isoform levels (Gapinske et al., 2018; Konermann et al., 2018; Thalhammer et al., 2018; Yuan et al., 2018), will help us to develop new and improved splicing therapies for brain disorders.

## Author Contributions

AT, FJ, and LC contributed jointly to the manuscript.

## Conflict of Interest

The authors declare that the research was conducted in the absence of any commercial or financial relationships that could be construed as a potential conflict of interest.

## Footnotes

^1^https://screen.wenglab.org/search/?q=CACNa1A&uuid=0&assembly=GRCh38

## Abbreviations

AMPAR: AMPA-type glutamate receptor
AS: alternative splicing
BDNF: brain-derived neurotrophic factor
CDF: Ca^2+^-dependent facilitation
CTCF: CCCTC-binding factor
GPCR: G protein-coupled receptor
mEPSCs: miniature excitatory postsynaptic currents
mGluR: metabotropic glutamate receptor
nSR100: Ser/Arg repeat-related protein of 100 kDa
nt: nucleotides
REST: repressor element 1 silencing transcription factor
SNP: single-nucleotide polymorphism
TTX: tetrodotoxin
UTR: untranslated region
VGCC: voltage-gated calcium channel.

## References

[B1] Andrade A. Denome S. Jiang Y. Q. Marangoudakis S. Lipscombe D. (2010). Opioid inhibition of N-type Ca^2+^ channels and spinal analgesia couple to alternative splicing. Nat. Neurosci. 13, 1249–1256. 10.1038/nn.2643 20852623 PMC2956429

[B2] Ango F. Prézeau L. Muller T. Tu J. C. Xiao B. Worley P. F.. (2001). Agonist-independent activation of metabotropic glutamate receptors by the intracellular protein Homer. Nature 411, 962–965. 10.1038/35082096 11418862

[B3] Babitch J. (1990). Channel hands. Nature 346, 321–322. 10.1038/346321b0 2165218

[B4] Baralle F. E. Giudice J. (2017). Alternative splicing as a regulator of development and tissue identity. Nat. Rev. Mol. Cell Biol. 18, 437–451. 10.1038/nrm.2017.27 28488700 PMC6839889

[B5] Bell T. J. Thaler C. Castiglioni A. J. Helton T. D. Lipscombe D. (2004). Cell-specific alternative splicing increases calcium channel current density in the pain pathway. Neuron 41, 127–138. 10.1016/s0896-6273(03)00801-8 14715140

[B6] Ben-Johny M. Yang P. S. Niu J. Yang W. Joshi-Mukherjee R. Yue D. T. (2014). Conservation of Ca^2+^/calmodulin regulation across Na and Ca^2+^ channels. Cell 157, 1657–1670. 10.1016/j.cell.2014.04.035 24949975 PMC4349408

[B7] Berke J. D. Paletzki R. F. Aronson G. J. Hyman S. E. Gerfen C. R. (1998). A complex program of striatal gene expression induced by dopaminergic stimulation. J. Neurosci. 18, 5301–5310. 10.1523/jneurosci.18-14-05301.1998 9651213 PMC6793476

[B8] Bottai D. Guzowski J. F. Schwarz M. K. Kang S. H. Xiao B. Lanahan A.. (2002). Synaptic activity-induced conversion of intronic to exonic sequence in Homer 1 immediate early gene expression. J. Neurosci. 22, 167–175. 10.1523/jneurosci.22-01-00167.2002 11756499 PMC6757601

[B9] Bourinet E. Soong T. W. Sutton K. Slaymaker S. Mathews E. Monteil A.. (1999). Splicing of α 1A subunit gene generates phenotypic variants of P- and Q-type calcium channels. Nat. Neurosci. 2, 407–415. 10.1038/8070 10321243

[B10] Brakeman P. R. Lanahan A. A. O’Brien R. Roche K. Barnes C. A. Huganir R. L.. (1997). Homer: a protein that selectively binds metabotropic glutamate receptors. Nature 386, 284–288. 10.1038/386284a0 9069287

[B11] Chaudhuri D. Chang S. Y. DeMaria C. D. Alvania R. S. Soong T. W. Yue D. T. (2004). Alternative splicing as a molecular switch for Ca^2+^/calmodulin-dependent facilitation of P/Q-type Ca^2+^ channels. J. Neurosci. 24, 6334–6342. 10.1523/jneurosci.1712-04.2004 15254089 PMC6729554

[B12] Chevenet F. Brun C. Bañuls A. L. Jacq B. Christen R. (2006). TreeDyn: towards dynamic graphics and annotations for analyses of trees. BMC Bioinformatics 7:439. 10.1186/1471-2105-7-439 17032440 PMC1615880

[B13] Cingolani L. A. Vitale C. Dityatev A. (2019). Intra- and extracellular pillars of a unifying framework for homeostatic plasticity: a crosstalk between metabotropic receptors and extracellular matrix. Front. Cell. Neurosci. 13:513. 10.3389/fncel.2019.00513 31803023 PMC6877475

[B15] Davis G. W. Müller M. (2015). Homeostatic control of presynaptic neurotransmitter release. Annu. Rev. Physiol. 77, 251–270. 10.1146/annurev-physiol-021014-071740 25386989

[B16] Day J. J. Sweatt J. D. (2010). DNA methylation and memory formation. Nat. Neurosci. 13, 1319–1323. 10.1038/nn.2666 20975755 PMC3130618

[B17] Diering G. H. Nirujogi R. S. Roth R. H. Worley P. F. Pandey A. Huganir R. L. (2017). Homer1a drives homeostatic scaling-down of excitatory synapses during sleep. Science 355, 511–515. 10.1126/science.aai8355 28154077 PMC5382711

[B18] Dubes S. Favereaux A. Thoumine O. Letellier M. (2019). miRNA-dependent control of homeostatic plasticity in neurons. Front. Cell. Neurosci. 13:536. 10.3389/fncel.2019.00536 31866828 PMC6906196

[B19] Eggermann E. Bucurenciu I. Goswami S. P. Jonas P. (2012). Nanodomain coupling between Ca^2+^ channels and sensors of exocytosis at fast mammalian synapses. Nat. Rev. Neurosci. 13, 7–21. 10.1038/nrn3125 22183436 PMC3617475

[B14] ENCODE Project Consortium. (2012). An integrated encyclopedia of DNA elements in the human genome. Nature 489, 57–74. 10.1038/nature11247 22955616 PMC3439153

[B20] Erickson M. G. Alseikhan B. A. Peterson B. Z. Yue D. T. (2001). Preassociation of calmodulin with voltage-gated Ca^2+^ channels revealed by FRET in single living cells. Neuron 31, 973–985. 10.1016/s0896-6273(01)00438-x 11580897

[B21] Fagni L. Worley P. F. Ango F. (2002). Homer as both a scaffold and transduction molecule. Sci. STKE 2002:re8. 10.1126/stke.2002.137.re8 12072556

[B22] Fernandes D. Carvalho A. L. (2016). Mechanisms of homeostatic plasticity in the excitatory synapse. J. Neurochem. 139, 973–996. 10.1111/jnc.13687 27241695

[B23] Flavell S. W. Greenberg M. E. (2008). Signaling mechanisms linking neuronal activity to gene expression and plasticity of the nervous system. Annu. Rev. Neurosci. 31, 563–590. 10.1146/annurev.neuro.31.060407.125631 18558867 PMC2728073

[B24] Flavell S. W. Kim T. K. Gray J. M. Harmin D. A. Hemberg M. Hong E. J.. (2008). Genome-wide analysis of MEF2 transcriptional program reveals synaptic target genes and neuronal activity-dependent polyadenylation site selection. Neuron 60, 1022–1038. 10.1016/j.neuron.2008.11.029 19109909 PMC2630178

[B25] Frank C. A. Kennedy M. J. Goold C. P. Marek K. W. Davis G. W. (2006). Mechanisms underlying the rapid induction and sustained expression of synaptic homeostasis. Neuron 52, 663–677. 10.1016/j.neuron.2006.09.029 17114050 PMC2673733

[B26] Furlanis E. Scheiffele P. (2018). Regulation of neuronal differentiation, function and plasticity by alternative splicing. Annu. Rev. Cell Dev. Biol. 34, 451–469. 10.1146/annurev-cellbio-100617-062826 30028642 PMC6697533

[B27] Gapinske M. Luu A. Winter J. Woods W. S. Kostan K. A. Shiva N.. (2018). CRISPR-SKIP: programmable gene splicing with single base editors. Genome Biol. 19:107. 10.1186/s13059-018-1482-5 30107853 PMC6092781

[B28] Gardill B. R. Rivera-Acevedo R. E. Tung C. C. Okon M. McIntosh L. P. Van Petegem F. (2018). The voltage-gated sodium channel EF-hands form an interaction with the III-IV linker that is disturbed by disease-causing mutations. Sci. Rep. 8:4483. 10.1038/s41598-018-22713-y 29540853 PMC5852250

[B29] Gehman L. T. Stoilov P. Maguire J. Damianov A. Lin C. H. Shiue L.. (2011). The splicing regulator Rbfox1 (A2BP1) controls neuronal excitation in the mammalian brain. Nat. Genet. 43, 706–711. 10.1038/ng.841 21623373 PMC3125461

[B30] Giuffrida R. Musumeci S. D’Antoni S. Bonaccorso C. M. Giuffrida-Stella A. M. Oostra B. A.. (2005). A reduced number of metabotropic glutamate subtype 5 receptors are associated with constitutive homer proteins in a mouse model of fragile X syndrome. J. Neurosci. 25, 8908–8916. 10.1523/JNEUROSCI.0932-05.2005 16192381 PMC6725593

[B31] Gonatopoulos-Pournatzis T. Wu M. Braunschweig U. Roth J. Han H. Best A. J.. (2018). Genome-wide CRISPR-Cas9 interrogation of splicing networks reveals a mechanism for recognition of autism-misregulated neuronal microexons. Mol. Cell 72, 510.e12–524.e12. 10.1016/j.molcel.2018.10.008 30388412

[B32] Graves T. D. Imbrici P. Kors E. E. Terwindt G. M. Eunson L. H. Frants R. R.. (2008). Premature stop codons in a facilitating EF-hand splice variant of CaV2.1 cause episodic ataxia type 2. Neurobiol. Dis. 32, 10–15. 10.1016/j.nbd.2008.06.002 18606230

[B33] Gray A. C. Raingo J. Lipscombe D. (2007). Neuronal calcium channels: splicing for optimal performance. Cell Calcium 42, 409–417. 10.1016/j.ceca.2007.04.003 17512586 PMC2001240

[B34] Hatje K. Rahman R. U. Vidal R. O. Simm D. Hammesfahr B. Bansal V.. (2017). The landscape of human mutually exclusive splicing. Mol. Syst. Biol. 13:959. 10.15252/msb.20177728 29242366 PMC5740500

[B35] Hermey G. Bluthgen N. Kuhl D. (2017). Neuronal activity-regulated alternative mRNA splicing. Int. J. Biochem. Cell Biol. 91, 184–193. 10.1016/j.biocel.2017.06.002 28591617

[B36] Holderith N. Lorincz A. Katona G. Rózsa B. Kulik A. Watanabe M.. (2012). Release probability of hippocampal glutamatergic terminals scales with the size of the active zone. Nat. Neurosci. 15, 988–997. 10.1038/nn.3137 22683683 PMC3386897

[B37] Hu J. H. Park J. M. Park S. Xiao B. Dehoff M. H. Kim S.. (2010). Homeostatic scaling requires group I mGluR activation mediated by Homer1a. Neuron 68, 1128–1142. 10.1016/j.neuron.2010.11.008 21172614 PMC3013614

[B38] Huntley M. A. Srinivasan K. Friedman B. A. Wang T. M. Yee A. X. Wang Y.. (2020). Genome-wide analysis of differential gene expression and splicing in excitatory neurons and interneuron subtypes. J. Neurosci. 40, 958–973. 10.1523/jneurosci.1615-19.2019 31831521 PMC6988999

[B39] Ibata K. Sun Q. Turrigiano G. G. (2008). Rapid synaptic scaling induced by changes in postsynaptic firing. Neuron 57, 819–826. 10.1016/j.neuron.2008.02.031 18367083

[B40] Irimia M. Weatheritt R. J. Ellis J. D. Parikshak N. N. Gonatopoulos-Pournatzis T. Babor M.. (2014). A highly conserved program of neuronal microexons is misregulated in autistic brains. Cell 159, 1511–1523. 10.1016/j.cell.2014.11.035 25525873 PMC4390143

[B41] Jakawich S. K. Nasser H. B. Strong M. J. McCartney A. J. Perez A. S. Rakesh N.. (2010). Local presynaptic activity gates homeostatic changes in presynaptic function driven by dendritic BDNF synthesis. Neuron 68, 1143–1158. 10.1016/j.neuron.2010.11.034 21172615 PMC3046391

[B42] Javier E. Soto L. Lipscombe D. (2019). Cell-specific exon methylation and CTCF binding in neurons regulates calcium ion channel splicing and function. bioRxiv [Preprint]. 10.1101/2019.12.15.876185 PMC7124252 32213287

[B43] Jeans A. F. van Heusden F. C. Al-Mubarak B. Padamsey Z. Emptage N. J. (2017). Homeostatic presynaptic plasticity is specifically regulated by P/Q-type Ca^2+^ channels at mammalian hippocampal synapses. Cell Rep. 21, 341–350. 10.1016/j.celrep.2017.09.061 29020622 PMC5643522

[B44] Kammermeier P. J. Worley P. F. (2007). Homer 1a uncouples metabotropic glutamate receptor 5 from postsynaptic effectors. Proc. Natl. Acad. Sci. U S A 104, 6055–6060. 10.1073/pnas.0608991104 17389377 PMC1851615

[B45] Kato A. Ozawa F. Saitoh Y. Hirai K. Inokuchi K. (1997). vesl, a gene encoding VASP/Ena family related protein, is upregulated during seizure, long-term potentiation and synaptogenesis. FEBS Lett. 412, 183–189. 10.1016/s0014-5793(97)00775-8 9257717

[B46] Kim M. S. Pinto S. M. Getnet D. Nirujogi R. S. Manda S. S. Chaerkady R.. (2014). A draft map of the human proteome. Nature 509, 575–581. 10.1038/nature13302 24870542 PMC4403737

[B47] Konermann S. Lotfy P. Brideau N. J. Oki J. Shokhirev M. N. Hsu P. D. (2018). Transcriptome engineering with RNA-targeting type VI-D CRISPR effectors. Cell 173, 665.e14–676.e14. 10.1016/j.cell.2018.02.033 29551272 PMC5910255

[B48] Korb E. Wilkinson C. L. Delgado R. N. Lovero K. L. Finkbeiner S. (2013). Arc in the nucleus regulates PML-dependent GluA1 transcription and homeostatic plasticity. Nat. Neurosci. 16, 874–883. 10.1038/nn.3429 23749147 PMC3703835

[B49] Lazarevic V. Schöne C. Heine M. Gundelfinger E. D. Fejtova A. (2011). Extensive remodeling of the presynaptic cytomatrix upon homeostatic adaptation to network activity silencing. J. Neurosci. 31, 10189–10200. 10.1523/jneurosci.2088-11.2011 21752995 PMC6623065

[B50] Leber S. L. Llenos I. C. Miller C. L. Dulay J. R. Haybaeck J. Weis S. (2017). Homer1a protein expression in schizophrenia, bipolar disorder and major depression. J. Neural Transm. 124, 1261–1273. 10.1007/s00702-017-1776-x 28815330

[B51] Letellier M. Elramah S. Mondin M. Soula A. Penn A. Choquet D.. (2014). miR-92a regulates expression of synaptic GluA1-containing AMPA receptors during homeostatic scaling. Nat. Neurosci. 17, 1040–1042. 10.1038/nn.3762 25017011

[B52] Lipscombe D. Andrade A. Allen S. E. (2013). Alternative splicing: functional diversity among voltage-gated calcium channels and behavioral consequences. Biochim. Biophys. Acta 1828, 1522–1529. 10.1016/j.bbamem.2012.09.018 23022282 PMC3625486

[B53] Luco R. F. Allo M. Schor I. E. Kornblihtt A. R. Misteli T. (2011). Epigenetics in alternative pre-mRNA splicing. Cell 144, 16–26. 10.1016/j.cell.2010.11.056 21215366 PMC3038581

[B740] Lopez Soto E. J. Lipscombe D. (2020). Cell-specific exon methylation and CTCF binding in neurons regulate calcium ion channel splicing and function. Elife 9:e54879. 10.7554/eLife.54879 32213287 PMC7124252

[B54] Mantuano E. Romano S. Veneziano L. Gellera C. Castellotti B. Caimi S.. (2010). Identification of novel and recurrent CACNA1A gene mutations in fifteen patients with episodic ataxia type 2. J. Neurol. Sci. 291, 30–36. 10.1016/j.jns.2010.01.010 20129625

[B55] Mark M. D. Maejima T. Kuckelsberg D. Yoo J. W. Hyde R. A. Shah V.. (2011). Delayed postnatal loss of P/Q-type calcium channels recapitulates the absence epilepsy, dyskinesia, and ataxia phenotypes of genomic Cacna1a mutations. J. Neurosci. 31, 4311–4326. 10.1523/JNEUROSCI.5342-10.2011 21411672 PMC3065835

[B56] Martinez-Ortiz W. Cardozo T. J. (2018). An improved method for modeling voltage-gated ion channels at atomic accuracy applied to human cav channels. Cell Rep. 23, 1399–1408. 10.1016/j.celrep.2018.04.024 29719253 PMC5957504

[B57] Matera A. G. Wang Z. (2014). A day in the life of the spliceosome. Nat. Rev. Mol. Cell Biol. 15, 108–121. 10.1038/nrm3742 24452469 PMC4060434

[B58] Matosin N. Fernandez-Enright F. Lum J. S. Engel M. Andrews J. L. Gassen N. C.. (2016). Molecular evidence of synaptic pathology in the CA1 region in schizophrenia. NPJ Schizophr. 2:16022. 10.1038/npjschz.2016.22 27430010 PMC4944906

[B59] Mauger O. Scheiffele P. (2017). Beyond proteome diversity: alternative splicing as a regulator of neuronal transcript dynamics. Curr. Opin. Neurobiol. 45, 162–168. 10.1016/j.conb.2017.05.012 28609697 PMC6689270

[B60] Meadows J. P. Guzman-Karlsson M. C. Phillips S. Holleman C. Posey J. L. Day J. J.. (2015). DNA methylation regulates neuronal glutamatergic synaptic scaling. Sci. Signal. 8:ra61. 10.1126/scisignal.aab0715 26106219 PMC4764068

[B61] Montes M. Sanford B. L. Comiskey D. F. Chandler D. S. (2019). RNA splicing and disease: animal models to therapies. Trends Genet. 35, 68–87. 10.1016/j.tig.2018.10.002 30466729 PMC6339821

[B62] Mori M. Konno T. Ozawa T. Murata M. Imoto K. Nagayama K. (2000). Novel interaction of the voltage-dependent sodium channel (VDSC) with calmodulin: does VDSC acquire calmodulin-mediated Ca^2+^-sensitivity? Biochemistry 39, 1316–1323. 10.1021/bi9912600 10684611

[B63] Nakamura Y. Harada H. Kamasawa N. Matsui K. Rothman J. S. Shigemoto R.. (2015). Nanoscale distribution of presynaptic Ca^2+^ channels and its impact on vesicular release during development. Neuron 85, 145–158. 10.1016/j.neuron.2014.11.019 25533484 PMC4305191

[B64] Nguyen T. M. Schreiner D. Xiao L. Traunmuller L. Bornmann C. Scheiffele P. (2016). An alternative splicing switch shapes neurexin repertoires in principal neurons versus interneurons in the mouse hippocampus. Elife 5:e22757. 10.7554/eLife.22757 27960072 PMC5213383

[B65] Norris A. D. Calarco J. A. (2012). Emerging roles of alternative pre-mRNA splicing regulation in neuronal development and function. Front. Neurosci. 6:122. 10.3389/fnins.2012.00122 22936897 PMC3424503

[B66] Pan Q. Shai O. Lee L. J. Frey B. J. Blencowe B. J. (2008). Deep surveying of alternative splicing complexity in the human transcriptome by high-throughput sequencing. Nat. Genet. 40, 1413–1415. 10.1038/ng.259 18978789

[B67] Pecoraro-Bisogni F. Lignani G. Contestabile A. Castroflorio E. Pozzi D. Rocchi A.. (2018). REST-dependent presynaptic homeostasis induced by chronic neuronal hyperactivity. Mol. Neurobiol. 55, 4959–4972. 10.1007/s12035-017-0698-9 28786015

[B68] Peterson B. Z. DeMaria C. D. Adelman J. P. Yue D. T. (1999). Calmodulin is the Ca^2+^ sensor for Ca^2+^ -dependent inactivation of L-type calcium channels. Neuron 22, 549–558. 10.1016/s0896-6273(00)80709-6 10197534

[B69] Peterson B. Z. Lee J. S. Mulle J. G. Wang Y. de Leon M. Yue D. T. (2000). Critical determinants of Ca^2+^-dependent inactivation within an EF-hand motif of L-type Ca^2+^ channels. Biophys. J. 78, 1906–1920. 10.1016/s0006-3495(00)76739-7 10733970 PMC1300784

[B70] Pohl M. Bortfeldt R. H. Grützmann K. Schuster S. (2013). Alternative splicing of mutually exclusive exons—a review. Biosystems 114, 31–38. 10.1016/j.biosystems.2013.07.003 23850531

[B71] Pozzi D. Lignani G. Ferrea E. Contestabile A. Paonessa F. D’Alessandro R.. (2013). REST/NRSF-mediated intrinsic homeostasis protects neuronal networks from hyperexcitability. EMBO J. 32, 2994–3007. 10.1038/emboj.2013.231 24149584 PMC3831314

[B72] Quesnel-Vallières M. Dargaei Z. Irimia M. Gonatopoulos-Pournatzis T. Ip J. Y. Wu M.. (2016). Misregulation of an activity-dependent splicing network as a common mechanism underlying autism spectrum disorders. Mol. Cell 64, 1023–1034. 10.1016/j.molcel.2016.11.033 27984743

[B73] Raingo J. Castiglioni A. J. Lipscombe D. (2007). Alternative splicing controls G protein-dependent inhibition of N-type calcium channels in nociceptors. Nat. Neurosci. 10, 285–292. 10.1038/nn1848 17293861 PMC3027493

[B74] Raj B. Blencowe B. J. (2015). Alternative splicing in the mammalian nervous system: recent insights into mechanisms and functional roles. Neuron 87, 14–27. 10.1016/j.neuron.2015.05.004 26139367

[B75] Raj B. O’Hanlon D. Vessey J. P. Pan Q. Ray D. Buckley N. J.. (2011). Cross-regulation between an alternative splicing activator and a transcription repressor controls neurogenesis. Mol. Cell 43, 843–850. 10.1016/j.molcel.2011.08.014 21884984

[B76] Rebola N. Reva M. Kirizs T. Szoboszlay M. Lorincz A. Moneron G.. (2019). Distinct nanoscale calcium channel and synaptic vesicle topographies contribute to the diversity of synaptic function. Neuron 104, 693.e9–710.e9. 10.1016/j.neuron.2019.08.014 31558350

[B77] Ronesi J. A. Collins K. A. Hays S. A. Tsai N. P. Guo W. Birnbaum S. G.. (2012). Disrupted Homer scaffolds mediate abnormal mGluR5 function in a mouse model of fragile X syndrome. Nat. Neurosci. 15, 431–440, S1. 10.1038/nn.3033 22267161 PMC3288402

[B78] Ruiz-Velasco M. Kumar M. Lai M. C. Bhat P. Solis-Pinson A. B. Reyes A.. (2017). CTCF-mediated chromatin loops between promoter and gene body regulate alternative splicing across individuals. Cell Syst. 5, 628.e6–637.e6. 10.1016/j.cels.2017.10.018 29199022

[B79] Schanzenbächer C. T. Langer J. D. Schuman E. M. (2018). Time- and polarity-dependent proteomic changes associated with homeostatic scaling at central synapses. Elife 7:e33322. 10.7554/eLife.33322 29447110 PMC5814146

[B80] Schanzenbächer C. T. Sambandan S. Langer J. D. Schuman E. M. (2016). Nascent proteome remodeling following homeostatic scaling at hippocampal synapses. Neuron 92, 358–371. 10.1016/j.neuron.2016.09.058 27764671 PMC5078608

[B81] Schaukowitch K. Reese A. L. Kim S. K. Kilaru G. Joo J. Y. Kavalali E. T.. (2017). An intrinsic transcriptional program underlying synaptic scaling during activity suppression. Cell Rep. 18, 1512–1526. 10.1016/j.celrep.2017.01.033 28178527 PMC5524384

[B82] Schreiner D. Nguyen T. M. Russo G. Heber S. Patrignani A. Ahrné E.. (2014). Targeted combinatorial alternative splicing generates brain region-specific repertoires of neurexins. Neuron 84, 386–398. 10.1016/j.neuron.2014.09.011 25284007

[B83] Schreiner D. Simicevic J. Ahrné E. Schmidt A. Scheiffele P. (2015). Quantitative isoform-profiling of highly diversified recognition molecules. Elife 4:e07794. 10.7554/eLife.07794 25985086 PMC4489214

[B84] Shepherd J. D. Rumbaugh G. Wu J. Chowdhury S. Plath N. Kuhl D.. (2006). Arc/Arg3.1 mediates homeostatic synaptic scaling of AMPA receptors. Neuron 52, 475–484. 10.1016/j.neuron.2006.08.034 17088213 PMC1764219

[B85] Shiraishi-Yamaguchi Y. Furuichi T. (2007). The homer family proteins. Genome Biol. 8:206. 10.1186/gb-2007-8-2-206 17316461 PMC1852408

[B86] Shukla S. Kavak E. Gregory M. Imashimizu M. Shutinoski B. Kashlev M.. (2011). CTCF-promoted RNA polymerase II pausing links DNA methylation to splicing. Nature 479, 74–79. 10.1038/nature10442 21964334 PMC7398428

[B87] Soong T. W. DeMaria C. D. Alvania R. S. Zweifel L. S. Liang M. C. Mittman S.. (2002). Systematic identification of splice variants in human P/Q-type channel α_1_2.1 subunits: implications for current density and Ca^2+^-dependent inactivation. J. Neurosci. 22, 10142–10152. 10.1523/JNEUROSCI.22-23-10142.2002 12451115 PMC6758771

[B88] Sutton M. A. Ito H. T. Cressy P. Kempf C. Woo J. C. Schuman E. M. (2006). Miniature neurotransmission stabilizes synaptic function *via* tonic suppression of local dendritic protein synthesis. Cell 125, 785–799. 10.1016/j.cell.2006.03.040 16713568

[B89] Szumlinski K. K. Kalivas P. W. Worley P. F. (2006). Homer proteins: implications for neuropsychiatric disorders. Curr. Opin. Neurobiol. 16, 251–257. 10.1016/j.conb.2006.05.002 16704932

[B90] Thalhammer A. Contestabile A. Ermolyuk Y. S. Ng T. Volynski K. E. Soong T. W.. (2017). Alternative splicing of P/Q-type Ca^2+^ channels shapes presynaptic plasticity. Cell Rep. 20, 333–343. 10.1016/j.celrep.2017.06.055 28700936

[B91] Thalhammer A. Jaudon F. Cingolani L. A. (2018). Combining optogenetics with artificial microRNAs to characterize the effects of gene knockdown on presynaptic function within intact neuronal circuits. J. Vis. Exp. 133:e57223. 10.3791/57223 29608168 PMC5931759

[B92] Treutlein B. Gokce O. Quake S. R. Südhof T. C. (2014). Cartography of neurexin alternative splicing mapped by single-molecule long-read mRNA sequencing. Proc. Natl. Acad. Sci. U S A 111, E1291–E1299. 10.1073/pnas.1403244111 24639501 PMC3977267

[B93] Turrigiano G. (2011). Too many cooks? Intrinsic and synaptic homeostatic mechanisms in cortical circuit refinement. Annu. Rev. Neurosci. 34, 89–103. 10.1146/annurev-neuro-060909-153238 21438687

[B94] Ullrich B. Ushkaryov Y. A. Südhof T. C. (1995). Cartography of neurexins: more than 1000 isoforms generated by alternative splicing and expressed in distinct subsets of neurons. Neuron 14, 497–507. 10.1016/0896-6273(95)90306-2 7695896

[B95] van Loo K. M. Schaub C. Pernhorst K. Yaari Y. Beck H. Schoch S.. (2012). Transcriptional regulation of T-type calcium channel CaV3.2: bi-directionality by early growth response 1 (Egr1) and repressor element 1 (RE-1) protein-silencing transcription factor (REST). J. Biol. Chem. 287, 15489–15501. 10.1074/jbc.m111.310763 22431737 PMC3346130

[B96] Vigues S. Gastaldi M. Massacrier A. Cau P. Valmier J. (2002). The α_1A_ subunits of rat brain calcium channels are developmentally regulated by alternative RNA splicing. Neuroscience 113, 509–517. 10.1016/s0306-4522(02)00213-0 12150771

[B97] Voineagu I. Wang X. Johnston P. Lowe J. K. Tian Y. Horvath S.. (2011). Transcriptomic analysis of autistic brain reveals convergent molecular pathology. Nature 474, 380–384. 10.1038/nature10110 21614001 PMC3607626

[B98] Vuong C. K. Black D. L. Zheng S. (2016). The neurogenetics of alternative splicing. Nat. Rev. Neurosci. 17, 265–281. 10.1038/nrn.2016.27 27094079 PMC4861142

[B99] Wang E. T. Sandberg R. Luo S. Khrebtukova I. Zhang L. Mayr C.. (2008). Alternative isoform regulation in human tissue transcriptomes. Nature 456, 470–476. 10.1038/nature07509 18978772 PMC2593745

[B100] Weyrer C. Turecek J. Niday Z. Liu P. W. Nanou E. Catterall W. A.. (2019). The role of Ca_V_2.1 channel facilitation in synaptic facilitation. Cell Rep. 26, 2289.e3–2297.e3. 10.1016/j.celrep.2019.01.114 30811980 PMC6597251

[B101] Xiao B. Tu J. C. Petralia R. S. Yuan J. P. Doan A. Breder C. D.. (1998). Homer regulates the association of group 1 metabotropic glutamate receptors with multivalent complexes of homer-related, synaptic proteins. Neuron 21, 707–716. 10.1016/s0896-6273(00)80588-7 9808458

[B102] Yeo G. Holste D. Kreiman G. Burge C. B. (2004). Variation in alternative splicing across human tissues. Genome Biol. 5:R74. 10.3390/ijms20163977 15461793 PMC545594

[B103] Yuan J. Ma Y. Huang T. Chen Y. Peng Y. Li B.. (2018). Genetic modulation of RNA splicing with a CRISPR-guided cytidine deaminase. Mol. Cell 72, 380.e7–394.e7. 10.1016/j.molcel.2018.09.002 30293782

[B104] Zhang Y. Chen K. Sloan S. A. Bennett M. L. Scholze A. R. O’Keeffe S.. (2014). An RNA-sequencing transcriptome and splicing database of glia, neurons, and vascular cells of the cerebral cortex. J. Neurosci. 34, 11929–11947. 10.1523/JNEUROSCI.1860-14.2014 25186741 PMC4152602

[B105] Zhao C. Dreosti E. Lagnado L. (2011). Homeostatic synaptic plasticity through changes in presynaptic calcium influx. J. Neurosci. 31, 7492–7496. 10.1523/JNEUROSCI.6636-10.2011 21593333 PMC3124754

[B106] Zuhlke R. D. Pitt G. S. Deisseroth K. Tsien R. W. Reuter H. (1999). Calmodulin supports both inactivation and facilitation of L-type calcium channels. Nature 399, 159–162. 10.1038/20200 10335846

